# Models of professional regulation: institutionalizing an agency relationship

**DOI:** 10.1186/2045-4015-2-10

**Published:** 2013-03-27

**Authors:** Carolyn Hughes Tuohy

**Affiliations:** 1 School of Public Policy and Governance, University of Toronto, Toronto, CANADA

## Abstract

The regulation of medical practice can historically be understood as a second-level agency relationship whereby the state delegated authority to professional bodies to police the primary agency relationship between the individual physician and the patient. Borow, Levi and Glekin show how different national systems vary in the degree to which they insist on institutionally insulating the agency function from the promotion of private professional interests, and relate these variations to different models of the health care state. In fact these differences have even deeper roots in different “liberal” or “coordinated” varieties of capitalist political economies. Neither model is inherently more efficient than the other: what matters is the internal coherence or logic of these systems that conditions the expectations of actors in responding to particular challenges. The territory that Borow, Levi and Glekin have usefully mapped invites further exploration in this regard.

This is a commentary on http://www.ijhpr.org/content/2/1/8.

## 

At the heart of the political economy of health care lies an agency relationship between the physician and the patient, whereby patients effectively delegate decisionmaking authority regarding diagnosis and treatment to the provider and trust her to act in the patient’s interest [1,2]. The advent of the internet as an alternative source of information and the rise of an agenda of “patient-centered” care have not diminished the centrality of this relationship – indeed, these forces have arguably reinforced its importance while subjecting it to new demands and stresses.

Like any agency relationship, the physician-patient connection is subject to inherent conflicts of interest, since it is bound up with the physician’s source of livelihood. Much of health care policy can be understood as a quest by the state to control these conflicts, and to harness the power of the relationship to public objectives. Initially, the objectives were to ensure that individual physicians had the appropriate knowledge and expertise to make diagnostic and treatment decisions, and that they exercised that knowledge in accordance with professional standards of behaviour. These judgments, however, required assessors who were themselves grounded in the professional knowledge base. In theory, the state could have employed physicians to perform this function. But in most advanced nations, the state chose instead to tap the expertise and professional credibility of pre-existing professional networks by delegating authority to professional bodies to regulate their members on behalf of the state - establishing what was in effect a “second-level” agency relationship ^a^.

Distinguishing between this agency role for professional bodies on the one hand, and the right of professionals to engage in collective action in their own interests (including pecuniary interests) on the other, has always presented an institutional challenge. The advent of third-party payment, either public or private, as the norm in most health care systems in the twentieth century sharpened this challenge by requiring physicians to engage in various forms of collective bargaining with payers.

In an illuminating review, Borow, Levi and Glekin [3] show how different national systems vary in the degree to which they insist on institutionally insulating the agency function from the promotion of private professional interests. They relate these national differences to the models of governance and finance that characterize the health care systems more broadly, using categories first developed by Moran [4]. Avatars of two models - one in which the market provides the centre of gravity for the health care system (the United States) and the other the “entrenched command-and-control” or state-based model (the United Kingdom) have each come to insist on a greater institutional separation of the agency role from the representation of private interest than have “corporatist” nations, with their more collaborative operating principles, as represented by Germany. “Hybrid” systems such as those of the Netherlands and Israel fall between these extremes, allowing professional associations charged with pursuing the private interests of their members to have some role in regulatory decision-making, though not as extensive a role as in the German case.

This characterization is useful in drawing attention to variation in the norms, both formal and informal, that govern the operation of the agency relationship between the medical professional and the state in advanced nations, and in relating this variation to broader health system characteristics and to historical factors. In this brief commentary I can offer only a few glosses on this contribution and its implications for the further development of public policy in this area.

First, the portraits of the American and British systems need some shading. The authors somewhat overstate the exclusion of medical associations from regulatory decision-making in the United States, by portraying the state medical boards (the licensing bodies) as “independent agencies” appointed by the governor. While technically correct, this portrayal neglects the fact that the history of the development of the licensing boards is one of progressive disentanglement from state medical societies – a process that varied considerably across states and remains incomplete. In about 20 states the state medical societies (federated within the American Medical Association, or AMA) continue to advise on or nominate members for appointment to the medical boards. As noted in the text of the Borow et al. article (though not in the summary table), moreover, the AMA is also involved in the establishment of the boards which accredit specialist physicians.

In the UK, the institutional insulation of the licensing and disciplinary function from the representation of professional interests is more fully realized. But this fact should not obscure the other avenues through which professional influence over licensing and discipline is exercised. The General Medical Council was dominated by representatives of professional societies and academic institutions (though not the British Medical Association) from its founding in 1898 until the 1970s, and only in the 2000s was a principle of equal representation of lay and medical members established.

My second observation concerns the need to look beyond the health care system to the broader political economy to explain the national differences mapped by Borow, Levi and Glekin. The nations they review can be seen as representing different “varieties of capitalism (VOC):” the US and the UK fall into the domain of “liberal market economies” (LMEs) characterized by competition and arm’s-length relationships, while Germany and the Netherlands are closer to a “coordinated market economy” (CME) model based on networks and collaboration [5]. (In the VOC literature, Israel has been assigned to the liberal market economy type [6]). Within these broad groupings, moreover, there are further variations: the British style of regulation has historically been more informal and flexible than that of the US [7]; and no aspect of Dutch public policy can be understood without recognizing the long-standing and pervasive tension between the cultural values of “solidarity” and “subsidiarity” [8]. Hence the distinctive national variants of professional regulatory institutions described by Borow et al. have deeper roots than a focus on the structure of the “health care state” alone would suggest. If anything, the need to tap professional expertise through agency relationships may have tempered these differences in the health care arena.

The key insight from the VOC literature, however, is that *neither* the “liberal” or the “coordinated” model of politico-economic organization is inherently more efficient than the other. Rather, what matters is the internal coherence or logic of these systems that conditions the expectations of actors in responding to particular challenges ^b^. In the arena of professional regulation, it is conceivable that public objectives could be served *either* by a strict formal separation of roles *or* by an acknowledgement and appropriate weighting of these different interests within trust-based networks. It is, in fact, an empirical question as to the conditions under which each model is likely to be effective. There appears, for example, to be no relationship between the regulatory model and the income of physicians: the US, the Netherlands and Germany stand out internationally for the high relative incomes of both GPs and specialists. An ambitious attempt by OECD researchers to assess the “efficiency” ^c^ of health care systems, moreover, ranks the Netherlands and Germany well ahead of the UK and the US, although well below other “liberal” nations such as Australia (which also place regulators at greater distance from the interest-based medical association) [9]. This is a field ripe for further inquiry, and Borow, Levi and Glekin have paved the way.

## Endnotes

^a^This is essentially an academic interpretation of the relationship. However, a similar interpretation is offered in the 1975 report of the Merrison Committee regarding the British General Medical Council (GMC), a body most of whose membership until then was chosen by various medical constituencies. “We have suggested that the regulation of the profession can be looked upon as a contract made between the public and the profession. It is important to understand in this context that the GMC is merely the instrument for the proper supervision of this contract and that it derives its authority, and indeed its being, from legislation” [10]. Moran [4] also draws attention to this passage.

^b^There is extensive debate about the definition and the implications of “institutional complementarity” in this regard [11].

^c^Their measure of efficiency relates health care expenditure to the distance between actual life expectancy at birth and that which would be expected on the basis of various social and economic factors.

## Competing interests

The author declares that she has no competing interests.

## Author’s information

Carolyn Hughes Tuohy is Professor Emeritus of Political Science and Senior Fellow in the School of Public Policy and Governance at the University of Toronto, and a Fellow of the Royal Society of Canada. She works in the area of comparative health and social policy and is the author of *Accidental Logics*: *the Dynamics of Change in the Health Care Arena in the United States*, *Britain and Canada* (Oxford University Press 1999). She is currently completely a study of strategies of policy change in health care in the US, the UK, the Netherlands and Canada in the past 20 years.

## Commentary on

Borow M, Levi B and Glekin, M: Regulatory tasks of national medical associations - international comparison and the Israeli case. Isr J of Health Policy Res 2013, 2:8.
